# Glioma immunoediting, a driver of tumor evolution, and the next battle for immunotherapy

**DOI:** 10.18632/oncotarget.27865

**Published:** 2021-01-05

**Authors:** Adam M. Sonabend, Roger Stupp, Catalina Lee-Chang, Hideho Okada

**Keywords:** immunoediting, glioma, glioblastoma, immunotherapy, resistance

Cancer immunoediting has been proposed as a mechanism where the adaptive and innate immunity sculpts the immunogenicity of developing tumors, selecting for tumor cell variants that can evade immune surveillance [1–3]. If anti-tumoral immunity is capable of recognizing some tumor cell variants and not others, cancer immunoediting likely influences the evolutionary path of the cancer.

Glioblastoma (GBM), the most common and malignant of all primary brain tumors in adults, is notorious for its immunosuppressive features, and for its resilience to immunotherapy. Cancer immunoediting relies on tumor genomic heterogeneity, a hallmark of GBM [4]. Whereas glioma immunoediting is a likely explanation for the immunosuppressive phenotype encountered in gliomas, investigation of its role during gliomagenesis is challenging. Based on observations and correlative studies the literature suggests the existence of glioma immunoediting [5–7]. Yet, investigation to determine whether the immune microenvironment influences glioma phenotype or genotype during tumor development, and whether this ultimately leads to selection of glioma features that favor evasion of anti-tumoral immunity, had not been explored until recently.

CD8+ T-cells are one of the most established anti-tumoral effector immune cells. Whereas at diagnosis GBM has scant to no CD8+ T-cell infiltration in the tumor due to among other things T-cell sequestration in the bone marrow, [8] the abundance and relevance of CD8+ T-cells in early stages of GBM development remains to be determined. We thus hypothesized that CD8+ T-cells subject gliomas to immunoediting, as these cytotoxic T-cells might deplete immunogenic tumor clones, selecting immune evasive and immune-suppressive tumor variants. A recent publication by Sonabend’s group [9] directly investigated whether CD8+ T-cells can influence glioma phenotype, tumor genetic alterations, and tumor immunogenicity using transgenic mouse models where tumors are generated *de novo*. By depleting CD8+ T-cells prior to and during development of transgenic murine gliomas, this study investigated how these T-cells influence tumor immunogenicity and progression. Upon transplantation, gliomas developed in the absence of CD8+ T-cells engrafted poorly in recipients with intact immunity, but readily grafted in hosts with CD8+ T-cell depletion. On the other hand, gliomas that developed in the presence of CD8+ T-cells engrafted well in immunocompetent mice. This implies that gliomas that develop without the pressure exerted by CD8+ T-cells might be more immunogenic. Moreover, these gliomas exhibited increased activation of MAPK signaling, mitoses, aneuploidy, gene fusions, upregulation of genes related to the cGAS-STING pathway, as well as more robust macrophage/microglia infiltration. This study revealed that CD8+ T-cells mediate glioma immunoediting (at least on this model), thus influencing the selection of genomic alterations and modulating the tumor microenvironment during gliomagenesis, leading to a non-immunogenic glioma phenotype.

Glioma immunoediting may also be highly relevant in a clinical-stage of disease. Growing evidence suggest that immune escape of poorly antigenic clones is likely a major mechanism that drives recurrence of GBM that had an initial response to immunotherapy. Radadan, Sonabend and collaborators reported the disappearance of immunogenic glioma cell variants following PD-1 checkpoint inhibition in recurrent GBM patients who initially responded to treatment, whereas on the same patients, mutations that were not predicted to be immunogenic, did not disappear [10]. Similarly, glioma immunoediting and antigenic escape may also be taking place in the context of targeted immunotherapy such as with chimeric antigen receptor (CAR) T-cell therapy. Clinical trials evaluating CAR T-cells reported the loss of the targeting antigen due to therapeutic selection of cells that do not express it [11–13]. Interestingly, one of these studies also revealed a post-treatment GBM immunosuppressive microenvironment that was not present before therapy, raising concerns of immunoediting as a mechanism of resistance to this treatment modality [12].

It is becoming increasingly clear that due to glioma immunoediting, immunotherapy designed to target one specific antigen will likely lead to tumor escape and lack of efficacy. Novel forms of immunotherapy that address intra-tumoral antigenic heterogeneity up-front are therefore warranted. Targeting multiple tumor antigens simultaneously might minimize the possibility of selection of clones that avoid the initial therapy in this context. However, there are not many glioma-specific antigens. If a regimen targeted multiple antigens that are not necessarily specific to glioma cells, it would likely induce on-target, off-tumor toxicity. To overcome this dilemma, in addition to local delivery of CAR T-cells in the glioma site, novel cell engineering strategies, such as a use of sequential CAR expression system, which would allow local expression of CAR against multiple antigens, may be adopted in for safe and effective treatment of antigenically heterogenous cancer, such as glioma.

In summary, the pre-clinical and clinical evidence supporting the existence of glioma immunoediting and its contribution to recurrence following immunotherapy is emerging. Novel strategies that directly address this issue upfront might maximize the chances of effective immunotherapy for GBM, a disease that has so far deemed immunologically “cold”.
